# Diminished Macrophage Apoptosis and Reactive Oxygen Species Generation after Phorbol Ester Stimulation in Crohn's Disease

**DOI:** 10.1371/journal.pone.0007787

**Published:** 2009-11-12

**Authors:** Christine D. Palmer, Farooq Z. Rahman, Gavin W. Sewell, Afshan Ahmed, Margaret Ashcroft, Stuart L. Bloom, Anthony W. Segal, Andrew M. Smith

**Affiliations:** 1 Department of Medicine, Centre for Molecular Medicine, University College London, London, United Kingdom; 2 Department of Gastroenterology, University College London Hospital, London, United Kingdom; 3 Department of Medicine, Centre for Cell Signalling and Molecular Genetics, University College London, London, United Kingdom; Charité-Universitätsmedizin Berlin, Germany

## Abstract

**Background:**

Crohn's Disease (CD) is a chronic relapsing disorder characterized by granulomatous inflammation of the gastrointestinal tract. Although its pathogenesis is complex, we have recently shown that CD patients have a systemic defect in macrophage function, which results in the defective clearance of bacteria from inflammatory sites.

**Methodology/Principal Findings:**

Here we have identified a number of additional macrophage defects in CD following diacylglycerol (DAG) homolog phorbol-12-myristate-13-acetate (PMA) activation. We provide evidence for decreased DNA fragmentation, reduced mitochondrial membrane depolarization, impaired reactive oxygen species production, diminished cytochrome c release and increased IL-6 production compared to healthy subjects after PMA exposure. The observed macrophage defects in CD were stimulus-specific, as normal responses were observed following p53 activation and endoplasmic reticulum stress.

**Conclusion:**

These findings add to a growing body of evidence highlighting disordered macrophage function in CD and, given their pivotal role in orchestrating inflammatory responses, defective apoptosis could potentially contribute to the pathogenesis of CD.

## Introduction

Crohn's disease (CD) is a chronic relapsing inflammatory disease of the gastrointestinal tract associated with considerable lifelong morbidity[1]. It is characterized by granulomatous inflammation that most frequently affects the terminal ileum and colon. The incidence of CD has risen dramatically since the latter part of the 20^th^ century for reasons that remain poorly understood[2].

Despite tremendous advances in our understanding of the immunology of the gastrointestinal tract, the pathogenesis of CD remains elusive and highly contentious. Patient heterogeneity supports the complex nature of this disease and is a major difficulty in defining its cause. Various hypotheses concerning the pathogenetic mechanisms have been proposed over the years[3]. Most implicate a dysregulated mucosal immune response to intestinal luminal contents in those with a susceptible immunological background. The etiology of CD is almost certainly multifactorial, with numerous genetic and environmental factors that differ between individuals giving rise to a common syndrome.

We have previously shown a failure of acute inflammation in CD that is systemic and operates at the level of the macrophage[4], [5]. This defect results in diminished pro-inflammatory cytokine release, reduced neutrophil recruitment and the persistence of bacterial products within the tissue, which can potentially drive chronic inflammation. Other groups have previously shown abnormal apoptosis in both neutrophils and T-lymphocytes from CD patients under a variety of conditions[6], [7], and both anti-TNF and 5-aminosalicylic acid (5-ASA) therapy have been shown to induce apoptosis in leukocytes from CD patients[8]–[10]. These observations lead us to investigate whether CD macrophages also exhibit an apoptotic defect which may contribute to the immuno-pathology of CD.

Apoptosis is a tightly-regulated mechanism in controlling tissue homeostasis that can be initiated by a variety of signals and stress factors; Its physiological and pathological importance is highlighted by the fact that dysregulated apoptosis underlies many cancers and malignancies[11]. Concurrently, it has been shown that CD can predispose to an increased risk of developing colorectal cancers[12]. Studies in mice showed that neutrophil and macrophage apoptosis were characteristics of the resolving phase of inflammation[13], suggesting an important role for apoptosis in the resolution of inflammation, which is defective in many chronic inflammatory diseases[14]. Induction of apoptosis can occur via extrinsic factors (through death domain-containing receptors) or via intrinsic factors such as activation of tumor suppressor protein p53, which is activated in response to DNA damage, UV radiation and a range of chemotherapeutic drugs, and induces apoptosis-regulating pathways involving the mitochondria[15], [16]. Such intrinsic factors also include reactive oxygen species (ROS), which was shown to induce apoptosis in RAW264.7 macrophages and are posited to function via the mitochondria[17], [18]. Furthermore, studies in murine hepatocytes have shown that ROS-induced apoptosis required mitochondrial involvement in a protein kinase C (PKC)-dependent manner[19]. PKCs are a group of kinases that have been widely associated with apoptotic signaling[20]. Studies have shown that the regulation of PKC activity is highly complex, involving both a variety of phosphorylation events at different amino acid residues and conformational changes/cleavages conveying different states of (de)activation, depending on isoform, cell type and stimulus[21]–[23]. In particular, novel isoforms PKCδ and PKCε have been implicated in regulating cell survival and apoptosis[22], by interacting with a variety of proteins from the apoptotic machinery, including mitochondria-associated genes and caspases during apoptotic signaling processes[24], [25].

In this study, we demonstrate that stimulation with the DAG-homologue PMA[22] induces an abnormal apoptotic response, reduced NADPH oxidase activation and elevated IL-6 secretion in macrophage from CD patients. These findings add to a growing body of evidence highlighting disordered macrophage function during the acute inflammatory response in CD, providing further insight about the pathogenesis of this chronic disorder.

## Materials and Methods

### Ethics Statement

These studies were approved by the Joint UCL/UCLH Committee for the Ethics of Human Research (project number 04/0324). Written informed consent was obtained from all volunteers.

### Patients

Patients with endoscopically- and histologically-proven CD were identified through the gastroenterology outpatient clinics at University College London Hospitals (UCLH). All patients had quiescent disease at time of venesection (Harvey-Bradshaw Activity ≤3). Healthy controls were identified through the Department of Medicine, University College London (UCL). No subject was studied more than once in each of the different sets of experiments.

### Macrophage Isolation, Culture and Stimulation

Peripheral venous blood was collected from subjects into syringes containing 5 U/ml heparin. Mononuclear cells were isolated by differential centrifugation (2000 rpm, 30 min) over Lymphoprep (Axis-Shield, Oslo, Norway) and macrophages differentiated as previously described[5]. Adherent cells were scraped on day 5 and re-plated at densities (10^6^ cells/ml) in X-Vivo-15 medium (Cambrex, MD, USA). Plated macrophages were incubated overnight at 37°C, 5% CO_2_, and then stimulated as appropriate. Stimuli used were PMA at 1 µg/ml, 1 µM RITA (2,5-bis (5-hydroxymethyl-2-thienyl) furan, obtained from the National Cancer Centre, Drug Therapeutic Program, Frederick MD (NSC-652287)), 1 µM Thapsigargin (Sigma-Aldrich, UK), and 2.5 mM *N*-Acetyl-L-cysteine (NAC) (Sigma-Aldrich, UK), heat-killed *E. coli* (HkEc), prepared as previously described (4) at a ratio of 2.5 HkEc/macrophage, 2 µg/ml Pam_3_CSK_4_ (P_3_C) (Alexis Biochemicals, San Diego), 200 ng/ml LPS (Alexis). 1 µg/ml Infliximab (Remicade®) anti-TNF neutralizing antibody, human recombinant TNF at 25 ng/ml (Calbiochem, CA, USA), and human recombinant IL-6 at 10 ng/ml (R&D Systems, Abingdon, UK). Inhibitors used were 1 µM PKC inhibitor Bisindolylmaleimide I (BIM), (Calbiochem) and 25 µM topoisomerase II inhibitor etoposide phosphate (Sigma-Aldrich, UK).

### MTT Cell Viability Assay

Cell viability was ascertained by MTT assay (Boehringer Ingelheim, Berkshire, UK). Briefly, 20 µl of 2.5 ng/ml MTT were added to each well and incubated for 18 hours (h) at 37°C, 5% CO_2_. Supernatants were discarded and 100 µl/well of lysis solution (90% Isopropanol, 0.5% sodium dodecyl sulphate (SDS), 0.04 M NH_4_Cl, 9.5% H_2_0) added to each well for 1 h at room temperature. The absorbance was read at 570 nm using a FLUOstar OMEGA microplate reader and software (BMG LABTECH Ltd., Aylesbury, UK).

### DNA Fragmentation Assay

Macrophages were stimulated and cells permeabilized in 0.1% Triton X-100/PBS with 2 µM propidium iodide (PI) (Sigma-Aldrich, UK) for 1 h in the dark. DNA fragmentation was assessed by flow cytometry as previously described [26] using a FACSCalibur flow cytometer (BD Biosciences, NJ, USA), and analysis performed using the Cellquest™ software. The proportion of DNA giving fluorescence below the G_1-0_ peak (gated as M1) was used as a measure of apoptosis.

### Beadlyte Cytokine Secretion Assays

The expression profile of a panel of cytokines in macrophage supernatants was measured using the Beadlyte Bio-Plex™ human cytokine assay (Bio-Rad Laboratories, Hemel Hempstead, UK), according to the manufacturer's instructions. Our assay was customized to detect and quantify IL-1ra, RANTES, IL-6, GM-CSF and MCP-1.

### ELISA

The concentrations of human IL-10 (PeproTech, Inc., NJ, USA), IL6 (BD Biosciences) and IL-8 (PeproTech) were determined by ELISA according to the manufacturers' instructions. Cytochrome C concentrations in cell lysates were quantified using the Quantikine® Human Cytochrome C Immunoassay (R&D Systems) according to the manufacturer's instructions. Absorbance was read and analyzed at 450 nm on a FLUOstar OMEGA microplate reader and software (BMG LABTECH Ltd., Aylesbury, UK).

### TNF Bioassay

Release of bioactive TNF was measured using a cytotoxicity bioassay (obtained from Prof. B. Beutler, The Scripps Institute, CA, USA) as previously described[27]. Serially diluted rhTNF (100–0 pg/ml) (Calbiochem) was used to determine the standard curve for the assay.

### Amplex® Red Reactive Oxygen Intermediate (ROI) Release Assay

Release of H_2_O_2_ by PMA-stimulated HC and CD macrophages was assessed by Amplex® Red fluorometric assay alongside a standard curve. Cells were plated in a 96-well flat-bottomed plate at a density of 10^5^ cells/well. For inhibitor studies, cells were pre-incubated with 1 µM inhibitor for 1 h. H_2_O_2_ production was measured at 37°C in the presence of 4 µM Amplex® Red (Molecular Probes), 0.1 U/ml horseradish peroxidase (HRP) (Sigma-Aldrich) and, where appropriate, 1 µg/ml PMA and 1 µM BIM in sterile PBS using the FLUOstar OMEGA microplate reader (BMG LABTECH Ltd.). Excitation was set at 544 nm and emission was set at 590 nm, with measurements taken at 30 sec intervals. Rate of H_2_O_2_ production per hour (nM/h) was calculated over the first seven minutes (exponential phase) using the FLUOstar OMEGA software (BMG LABTECH Ltd.).

### Mitochondrial Membrane Potential Detection Assay

Loss of mitochondrial membrane potential in CD and HC macrophages was measured by flow cytometry using the APO LOGIX™ JC-1 kit (Peninsula Laboratories, LLC, CA, USA) according to the manufacturer's instructions. Fluorescence was measured using FACSCalibur flow cytometer (BD Biosciences, NJ, USA), and analysis performed using the Cellquest™ software.

### Statistical Analysis

Data were analyzed using paired or unpaired t-test using the GraphPad Prism 5 software.

## Results

### Abnormal PMA-Induced Cell Death Is Associated with Macrophages from CD Patients

**Figure 1:**
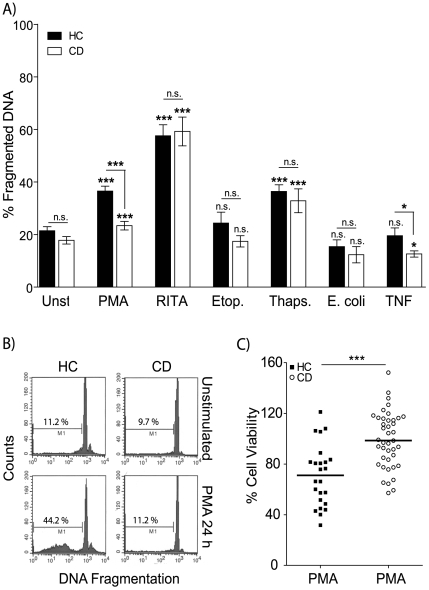
Defective apoptosis in macrophages from CD subjects. **A** Macrophages from HC and CD patients were untreated or stimulated with 1 µg/ml PMA (HC n = 39, CD n = 44), 1 µM RITA (HC n = 10, CD n = 7), 25 µM Etoposide (HC n = 7, CD n = 7), 1 µM Thapsigargin (HC n = 9, CD n = 8) for 24 h, *E. coli* (HC n = 10, CD n = 12) or 25 ng/ml recombinant TNF. DNA fragmentation (events below the G_1-0_ peak) was assessed by flow cytometry. Mean percentage ± SEM of apoptosis for HC (black bars) and CD (open bars) are shown (^o^ significance between stimulated and untreated, * between HC and CD). **B** Representative histograms for HC unstimulated (upper left panel), HC plus PMA 24 h (lower left), CD unstimulated (upper right) and CD plus PMA 24 h (lower right). **C** Viability for CD and HC macrophages following stimulation with 1 µg/ml PMA for 24 h was assessed by MTT assay. Data are presented as percent of untreated cells for HC (black squares, n = 24) and CD (open circles, n = 41) with group mean. *Statistical analysis:* Paired or unpaired t-test. *Symbols*: p<0.05 (* or ^o^), p<0.001 (*** or ^ooo^).

Abnormal apoptosis has been previously described in neutrophils and T lymphocytes isolated from CD patients[6], [7], [28]–[30]. We wanted to investigate the affects of numerous apoptotic stimuli on macrophages as these cells have been shown to be defective in patients with CD[5], [4]. Cell survival was determined by measuring the amount of DNA fragmentation before and after stimulation. In contrast to a published report on T lymphocytes apoptosis levels, baseline rate of DNA fragmentation was not significantly different between CD and HC macrophages (Figure 1). Macrophages were stimulated for 24 h with an panel of apoptosis-inducing agents: RITA (p53-activating small molecule)[31], [32], PMA, etoposide (topoisomerase II inhibitor), thapsigargin (sacro/endoplasmic reticulum (ER) Ca^2+^ ATPase inhibitor, induces ER stress), bacterial stimulation or TNF (Figure 1A). Increased DNA fragmentation was observed after RITA, PMA and thapsigargin stimulation in macrophages from HC and CD. Macrophages from CD patients were more resistant to PMA-induced DNA fragmentation (23.4±10.7 %) compared to those from HC (36.5±11.6 %, p<0.0001) (Figure 1A and B). The addition of TNF resulted in a moderate decrease in DNA fragmentation in macrophages from CD subjects (13±1.1 %) compared to unstimulated cells (15.4±2 %, p<0.05) and TNF treated HC macrophages (20±3 %, p<0.05). Etoposide and bacteria exposure had no effect on the level of DNA fragmentation compared to unstimulated macrophages. Macrophages from CD patients therefore undergo PMA-induced apoptosis, but the level of DNA fragmentation is only 64±4.4 % of that seen in HC. Decreased rate of apoptosis in PMA-stimulated CD macrophages was independent of disease phenotype (Figure S1), age (R^2^ = 0.03) and gender (p = 0.1399) (Table S1). These data show that the induction of apoptosis via p53 and ER stress pathways are unaffected in macrophages from CD patients. In contrast, abnormal macrophage apoptosis levels are evident after stimulation with PMA and to a lesser extent TNF.

In order to determine if decreased PMA-induced DNA fragmentation in macrophages from CD patients was the result of alterations in cell viability, a MTT assay was performed (Figure 1C). Macrophage survival 24 h after PMA stimulation was significantly higher in CD (99.2±3.4 %) than in HC (70.9±4.9 %, p<0.0001). These data show that macrophages from CD patients are much more resistant to PMA-induced apoptosis resulting in increased survival compared to HC. These results contrast with our findings in patients with chronic granulomatous disease (CGD)[33], who show normal PMA-induced macrophage apoptosis despite frequently presenting with granulomatous enterocolitis indistinguishable from CD [34], [35]. In addition, macrophages from patients with ulcerative colitis demonstrate a decrease in viability after PMA exposure (78.8 ±4.6 %, n = 13) that was equivalent to HC (p = 0.29) and significantly different to CD (p = 0.018) (Figure S2). Defective macrophage viability after PMA stimulation does not seem to be a consequence of general chronic inflammation, but specific to patients with CD.

### Loss of Mitochondrial Membrane Potential Is Impaired in PMA-Stimulated CD Macrophages

**Figure 2:**
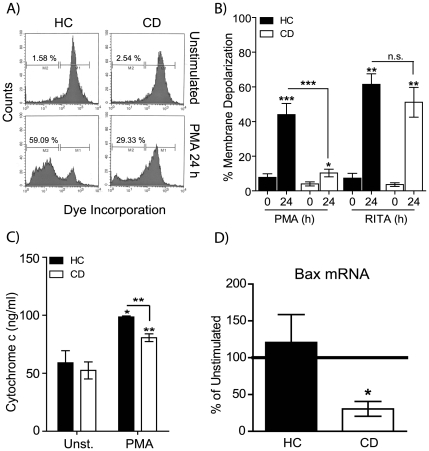
Mitochondrial membrane depolarization, cytochrome c and BAX expression are abnormal in CD macrophages after PMA activation. Macrophages were stimulated with 1 µg/ml PMA for 24 h and the effects on mitochondrial membrane potential, cytochrome c and BAX expression determined. **A** Representative histograms of macrophage population for HC unstimulated (upper left panel), HC PMA 24 h (lower left), CD unstimulated (upper right) and CD PMA 24 h (lower right) are shown. Gated populations show cells with an intact mitochondrial membranes (M1) and cells which have lost mitochondrial membrane integrity (M2). **B** Proportion of macrophages with mitochondrial membrane depolarization are shown as mean percentage ± SEM after either PMA (HC n = 9, CD n = 10) or RITA (HC n = 5, CD n = 5) stimulation. **C** Intracellular cytochrome C levels in macrophages stimulated with 1 µg/ml PMA for 24 h were measured by ELISA. Cytochrome C production (ng/ml) at 24 h is shown for HC (n = 4, black bars) and CD (n = 8, open bars). **D** Macrophages were stimulated with 1 µg/ml PMA for 4 h followed by total RNA isolation. Bax mRNA levels were determined by qPCR and expressed as the change in expression compared to unstimulated cells. *Statistical analysis:* Paired or unpaired t-test. *Symbols*: p<0.05 (*), p<0.01 (**), p<0.001 (***).

Cell death via the apoptotic pathway results from a number of key steps which include permeabilization of the mitochondrial membrane and loss of membrane potential, cytochrome C release and caspase 3 activation[36]. Ultimately these lead to DNA fragmentation, membrane blebbing and apoptosis. Loss of mitochondrial membrane potential in macrophages from CD and HC following PMA stimulation for 24 h was measured (Figure 2A and B). A significant loss of mitochondrial membrane potential after PMA exposure was evident in both HC (p<0.001) and CD (p<0.05) macrophages. The mean percentage of macrophages demonstrating loss of mitochondrial membrane potential was significantly lower in CD patients (10.3±2.2 %) compared to HC (44.0±6.4, p<0.01) (Figure 2A and B). Macrophages were incubated with RITA in order to investigate p53 signaling which also results in loss of mitochondrial membrane potential during the induction of apoptosis (Figure 2B)[31], [15], [32]. Stimulation with RITA induced a loss of mitochondrial membrane potential in macrophages from both HC (p<0.01) and CD (p<0.01), with no significant difference between the two groups (p = 0.3553). These findings further supporting the concept that the intrinsic apoptotic pathway downsteam of p53 is able to operate normally in macrophage from CD patients. Loss of mitochondrial membrane potential results in the release of cytochrome C[16], which was measured after stimulation. Intracellular cytochrome C levels in macrophages from CD were significantly lower than those in HC macrophages after PMA stimulation (p<0.01, Figure 2C). Resistance to apoptosis in mucosal T lymphocytes from CD patients has previously been shown to correlate with decreased Bax expression[24]. We therefore assessed Bax mRNA levels in HC and CD macrophages following PMA stimulation by quantitative PCR. PMA exposure resulted in the upregulation of Bax in HC macrophages (Figure 2D). The upregulation of Bax was significantly lower in macrophages from CD patients after PMA stimulation compared to HC subjects (Figure 2D). These results are consistent with the diminished apoptosis observed thus far, and provide further evidence for a general dysregulation of PMA-induced responses in macrophages from CD patients.

### Defective PMA-Induced Reactive Oxygen Species (ROS) Production in CD Macrophages

**Figure 3:**
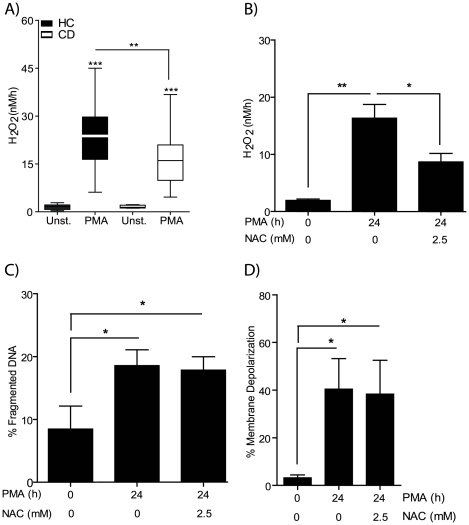
Decreased PMA-induced reactive oxygen species production in CD macrophages. NADPH oxidase activity was assessed by measuring the generation of H_2_O_2_ by macrophages from HC and CD subjects after stimulation with 1 µg/ml PMA. **A** Production of H_2_O_2_ (nM/h) was elevated after PMA stimulation in both HC (n = 29) and CD (n = 47) groups. Macrophages from CD patients demonstrated reduced H_2_O_2_ release than HC. **B** H_2_O_2_ levels were determined after HC macrophages (n = 4) pre-incubated with 2.5 mM *N*-Acetyl-L-cysteine (NAC) for 1 h followed by PMA stimulation. The presence of NAC resulted in reduced H_2_O_2_ levels. **C** DNA fragmentation and **D** mitochondrial membrane potential were unaltered by the presence of NAC. *Statistical analysis:* Paired or unpaired t-test. *Symbols*: p<0.05 (*), p<0.01 (**), p<0.001 (***), HC (black bars), CD (white bars).

In addition to an apoptotic response, PMA stimulation also induces the production of ROS in macrophages through the activation of the NADPH oxidase system[37]. PMA-induced ROS production, determined by H_2_O_2_ generation, in HC and CD macrophages was assessed (Figure 3A). The rate of H_2_O_2_ production was significantly decreased in CD macrophages (17.1±1.4 nM/h) compared to HC (23.7±1.9 nM/h, p<0.01). In order to assess whether there was a causal link between reduced H_2_O_2_ production and decreased apoptosis in macrophages from CD patients in response to PMA, cells were treated with the antioxidant *N*-acetyl-L-cysteine (NAC). Macrophages from HC pre-treated with NAC reduced the amount of free H_2_O_2_ after PMA-stimulation by 46.7 %, which was even greater than the 17% observed with macrophages from CD patients (Figure 3B). However, reduced H_2_O_2_ levels in the presence of NAC had no effect on PMA-induced DNA fragmentation (Figure 3C) or mitochondrial membrane potential (Figure 3D). This indicates that the decreased ROS levels observed in macrophages from CD patients are not associated with, and consequently not responsible for the resistance to PMA induced loss in cell viability.

Macrophages from CD patients demonstrate two distinct abnormalities after PMA stimulation: increased resistance to apoptosis and diminished NADPH oxidase activity. These results are in line with our previous findings showing normal levels of PMA-induced apoptosis in macrophages from CGD patients who generate no ROS due to a complete absence in NADPH oxidase activity[33].

### Increased IL-6 Production in Macrophages from CD Patients in Response to PMA

**Figure 4:**
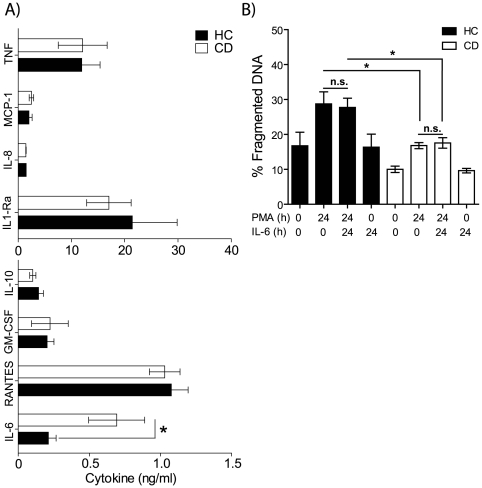
Dysregulated PMA-induced IL-6 production in CD macrophages. **A** Cytokine secretion from HC (n = 7) and CD (n = 10) macrophages following stimulation with 1 µg/ml PMA for six hours was assessed. IL-6 production was significantly elevated in macrophages from CD patients compared to HC subjects. **B** Measuring the effect of elevated IL-6 (1 ng/ml) on DNA fragmentation in the presence or absence of PMA stimulation. DNA fragmentation was unaltered by the inclusion of IL-6 in both HC (n = 7) and CD (n = 7) macrophages. *Statistical analysis:* Paired or unpaired t-test. *Symbols*: p<0.05 (*), p<0.01 (**), p<0.001 (***).

Previously, we have reported a defective acute inflammatory response to microbial challenge associated with macrophages from CD patients[5], [4]. We were therefore interested in determining the effects of chronic activation and reduced apoptosis with the DAG homolog-PMA on cytokine generation by macrophages from CD subjects. Analyses of cytokines produced in response to PMA did not reveal the same dramatic difference in cytokine release seen with bacterial and toll-like receptor stimulation [5]: TNF, MCP-1, IL-8, IL-1Ra, IL-10, GM-CSF and RANTES were released at levels not significantly different from HC macrophages (Figure 4A). Macrophages from CD patients produced significantly more IL-6 than HC at 24 h after PMA stimulation (Figure 4A, p<0.05). Increased secretion of IL-6 by CD macrophages after PMA stimulation is consistent with the elevated serum levels previously reported for patients with active disease[38]. There is also a direct correlation between IL-6 serum levels and disease severity[39]. IL-6 has also been shown to produce anti-apoptotic effects via gp130 and the activation of the JAK/STAT3 pathway[40]. In order to ascertain whether elevated IL-6 secretion exerted any effect on the apoptotic response, HC and CD macrophages were stimulated with recombinant IL-6 in the presence or absence of PMA. Addition of IL-6 to HC and CD macrophages did not alter the basal level of apoptosis seen in unstimulated cells (Figure 4B). As shown previously, the macrophages from CD subjects were more resistant to DNA fragmentation than HC after PMA activation. The inclusion of IL-6 had no effect on the rate of DNA fragmentation in either the HC or CD subjects with or without PMA. STAT3 phosphorylation was also similar between the two groups (data not shown). It therefore seems unlikely that the elevation in IL-6 is responsible for the reduced apoptosis in macrophages from CD patients.

### PMA Induced Apoptosis and ROS Generation Signals through a Bisindolylmaleimide Sensitive Pathway

**Figure 5:**
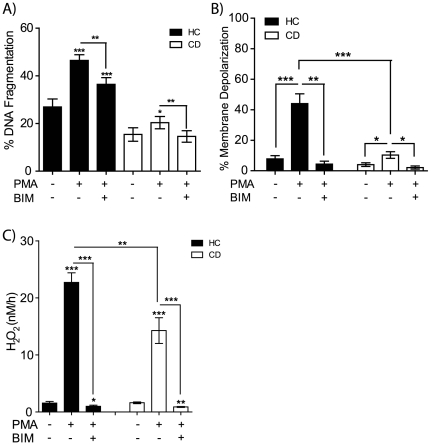
PMA induced apoptosis, NADPH oxidase activity and mitochondrial membrane depolarization are all inhibited by Bisindolylmaleimide I. Macrophages from HC (n = 5–10) and CD (n = 5–10) were left untreated, or pre-incubated with 1 µM Bisindolylmaleimide I (BIM) for 1 h followed by PMA stimulation. BIM significantly reduced **A** DNA fragmentation, **B** mitochondrial membrane depolarization and C H_2_O_2_ release in both HC and CD. Paired or unpaired t-test. *Symbols*: p<0.05 (*), p<0.01 (**), p<0.001 (***), HC (black bars), CD (white bars).

PMA has been previously shown to activate a number of intracellular signaling molecules which are sensitive to DAG generation downstream of phospholipase C. These include the classical and novel protein kinase C (PKC) family as well as a host of other DAG responsive molecules[22]. The effects on apoptosis and ROS generation in the presence of bisindolylmaleimide I (BIM), a potent but none-selective inhibitor of PKC, were assessed[41]. Pre-incubation with BIM significantly inhibits PMA-induced DNA fragmentation (Figure 5A), mitochondrial membrane depolarization (Figure 5B) and ROS generation (Figure 5C) in macrophages from HC and from CD subjects. The exact PMA sensitive molecules responsible for the abnormalities are still unknown, but our findings suggest that the abnormalities in macrophages from CD are downstream of a BIM-sensitive PMA inducible pathway.

## Discussion

This study identified several defects associated with macrophages from CD patients: 1) Macrophages show increased viability and decreased apoptosis downstream of the DAG homolog PMA. 2) The impaired apoptotic response is PMA-specific, as activation of apoptosis via p53 and ER stress are normal in CD macrophages. 3) PMA-induced NADPH oxidase activity is also impaired in CD macrophages. 4) PMA exposure also results in elevated IL-6 release. In addition to these observations we have recently described a major defect in the innate immune response of CD patients in response to bacterial stimulation, which results in impaired clearance as a consequence of defective cytokine secretion from macrophages[5]. We therefore propose that defective macrophage function plays a major role in the abnormal acute inflammatory response and subsequent chronic granulomatous inflammation in CD.

Studies investigating apoptosis in CD have thus far concentrated on lymphocytes and neutrophils[7], [6], [30], [29], [28], and this is the first time that a defect in CD macrophage apoptosis has been shown. Interestingly, aberrant apoptosis in T-lymphocytes was observed in response to numerous apoptotic stimuli, including IL-2 deprivation, Fas ligand binding, and nitric oxide (NO) exposure, as well as lower spontaneous apoptosis in CD biopsy tissue explants[30]. These data contrast with our findings on several accounts. Firstly, baseline (spontaneous) macrophage apoptosis was comparable in HC and CD subjects. Secondly, apoptotic responses following p53 activation and ER stress induction were normal, suggesting a more specific defect in macrophages compared to T lymphocytes in CD. These cell-type-specific differences are further highlighted by the fact that CD neutrophils exhibit delayed apoptosis in suspension and accelerated apoptosis following adhesion to fibronectin[30], and support our hypothesis of CD as a multi-factorial syndrome that has multiple interacting cellular and tissue components[5], [42].

PMA acts through the activation of DAG responsive proteins and targets a number of potential pathways in human macrophages. Protein kinase C (PKC) family members are especially sensitive to PMA activation and have been implicated in the induction of both apoptosis as well as NADPH oxidase activity[43], [44]. The conventional or classical PKCs (*α*, *β*I, *β*II and *γ*) and the novel PKCs (*δ, ε*, *η* and *θ*) are all activated by the additional binding of DAG or its homologue PMA[44]. Due to this complexity we were unable to identify abnormal PKC activity using isotype specific activation antibodies, but the inclusion of BIM, a non-selective PKC inhibitor provided some evidence to support their involvement. Furthermore, abnormal PKC activity has previously been identified in CD patients during acute inflammation[45]. Defective apoptosis and NADPH oxidase activity may result from abnormal PKC activity but equally it may depend on downstream events. The fact that apoptosis occurs normally when p53 and ER stress are activated directly suggest that the machinery required for programmed cell death functions normally in CD macrophages. Further work is needed to identify the specific PMA inducible defects in macrophages from CD patients.

We have previously shown that macrophages from CGD patients that completely lack NADPH oxidase activity apoptose normally after PMA activation[33]. The inclusion of the ROS sequester NAC resulted in decreased levels of H_2_O_2_ but had no apparent effect on the apoptotic response in macrophages from HC. These observations suggest that reduced NADPH oxidase activity does not confer protection against apoptosis after PMA stimulation in primary human macrophages.

We have also shown dysregulation of PMA-induced secretion of IL-6, a cytokine which has previously been associated with the chronic phase of CD[38], [46]. Macrophages are thought to be one of the main cell types responsible for elevated intestinal IL-6 levels[47], and although our results show that IL-6 production does not have an autocrine effect on macrophage apoptosis, it is nonetheless possible that increased IL-6 levels in active CD contributes to the pathogenesis. Current therapies for CD are being developed that specifically target IL-6 and its receptor gp130 and the results from these studies will help to determine the precise role IL-6 plays in the pathophysiology of CD[48]. Our previous research has clearly shown that microbial stimulation resulted in significantly reduced pro-inflammatory cytokine secretion, including IL-6, in patients with CD[4], [5]. This defect results in reduced neutrophil recruitment and the retention of bacteria within the tissue. It is plausible that chronic inflammation in CD is driven by the residual bacteria/bowel content in combination with defective macrophage apoptosis. This could result in the persistence of the pro-inflammatory stimuli, prolonged cytokine secretion, a failure in resolution, defective wound healing, granulomatous tissue formation, angiogenesis, fibrosis and scar formation; all of which are hallmarks of the chronic inflammatory phase in CD.

The precise mechanisms involved in acute inflammation and its subsequent resolution remain poorly understood, although it has become apparent that apoptosis plays an important role in the resolution phase. Initial work identified neutrophil apoptosis as a critical factor in switching off inflammation and inducing the resolution phase[49]. More recently, a role for macrophage apoptosis in the resolution phase of acute inflammation has been described[13]. It now seems that the induction and subsequent resolution of an acute inflammatory response require complex coordinated phases with regards to cellular recruitment and clearance of inflammatory cells. It is highly probable that defects affecting these processes contribute to the onset of human inflammatory diseases and specifically CD.

The importance of defective apoptosis in the immuno-pathology of CD is further substantiated by evidence that several efficacious CD therapies have the potential to induced apoptosis. Several different TNF-antagonists show clinical efficacy in inflammatory diseases[50]. These can broadly be divided into neutralizing antibodies (infliximab and adalimumab) and recombinant receptors (etanercept). Whilst both classes are equally clinically efficacious in rheumatoid arthritis, the recombinant TNF receptor/immunoglobulin G fusion protein etanercept is not effective in CD[51]. Studies in peripheral and lamina propria T lymphocytes have attributed this to the fact that, whilst both classes therapeutics neutralize TNF *in vitro*, only the neutralizing antibodies are capable of inducing apoptosis in these cells[8], [50], [52]. It has thus been proposed that the beneficial effects of anti-TNF therapies in active CD relate not to direct binding and sequestering soluble TNF, but cross-linking of membrane-bound forms and induction of leukocyte apoptosis[50]. Thiopurines and methotrexate are immunosuppresants widely used in the treatment of moderate to severe CD and other chronic inflammatory conditions. Thiopurines have been shown to induce apoptosis via induction of a mitochondrial pathway[53]. Methotrexate has been shown to induce apoptosis as well as elevating ROS generation[54], [55]. There is also evidence that 5-ASAs, another commonly used group of drugs used in mild CD, may have the ability to induced apoptosis in leukocytes[10], [56], [57]. Probiotics have recently attracted much interest as a potential treatment for CD. A recent study has shown that the administration of the probiotic *Lactobacillus casei* resulted in an increase in the number of intestinal lymphocytes undergoing apoptosis in active CD[58]. Collectively, these observations suggest that the clinical efficacy of commonly used CD therapies might at least in part be due to restoration of the apoptotic responses in macrophages and other leukocytes. The association between therapeutic success in CD and activation of apoptosis continue to increase and may be an important consideration in future drug development for this chronic inflammatory disease.

## Supporting Information

Table S1 Patient demographics. All the CD patients used in this study have been listed with gender, age, ethnicity, phenotype, current treatment and smoking status if known. m  =  male, f  =  female, TI  =  terminal ileal, MTX  =  methotrexate, *  =  data not available.
(0.01 MB PDF)

Figure S1 Altered apoptotic response in CD macrophages is independent of disease location. Data from CD macrophages presented in Figure 1B are presented as percent of apoptotic cells for each donor (HC n = 39; CD n = 44) sub-divided into disease location: colonic disease (col) (n = 15), ileocolonic disease (I/C) (n = 18) and terminal ileal disease (TI) (n = 11). Statistical analysis: Unpaired t-test. Symbols: p<0.001 (***), HC (black squares), col CD (open triangles), I/C CD (grey triangles) and TI CD (black triangles).
(1.29 MB EPS)

Figure S2 Abnormal macrophage response to PMA stimulation is specific for CD patients. Viability assay for macrophages from CD, HC and ulcerative colitis subjects following stimulation with PMA for 24 h. Data are presented as percent of untreated cells for CD (blue, n = 41), HC (red, n = 24) and UC (black, n = 13). Statistical analysis: Unpaired t-test.
(0.03 MB JPG)
