# Supplementary material for: Diminished Macrophage Apoptosis and Reactive Oxygen Species Generation after Phorbol Ester Stimulation in Crohn's Disease
Source: PLoS One. 2009 Nov 12;4(11):e7787. doi: 10.1371/journal.pone.0007787 (PMC2771353; doi:10.1371/journal.pone.0007787)
Supplement: Table S1 — Patient demographics. All the CD patients used in this study have been listed with gender, age, ethnicity, phenotype, current treatment and smoking status if known. m = male, f = female, TI = terminal ileal, MTX = methotrexate, * = data not available. (0.01 MB PDF) [file pone.0007787.s001.pdf]

Gender	Age	Ethnicity	Disease Location	Rx	Smoker
M	35	Caucasian	TI	5-ASA	N
F	34	Caucasian	colonic	5-ASA	N
F	23	Caucasian	colonic	5-ASA	N
F	29	Caucasian	ileocolonic	none	*
M	26	Caucasian	ileocolonic	none	N
F	70	Afro-carribean	ileocolonic	5-ASA	N
F	23	Caucasian	colonic, perianal	none	N
F	77	Caucasian	ileocolonic	5-ASA	N
M	28	Caucasian	ileocolonic, fistula	6-mercaptopurine	*
M	62	Caucasian	ileocolonic	none	*
M	32	Caucasian	TI	5-ASA	*
M	73	Caucasian	ileocolonic	Aspirin	*
M	29	Caucasian	colonic	5-ASA, AZA	N
F	37	Caucasian	colonic	5-ASA	*
M	38	Caucasian	ileocolonic	*	*
M	59	Caucasian	ileocolonic	5-ASA	*
M	26	Caucasian	ileocolonic		*
M	59	Caucasian	ileocolonic	none	N
F	*	Caucasian	ileocolonic	none	*
F	25	Caucasian	colonic	5-ASA	*
F	31	Caucasian	TI	*	*
M	37	Caucasian	TI	5-ASA	*
M	34	Caucasian	colonic	5-ASA, MTX, Ciprofloxacin	*
M	25	Caucasian	TI	5-ASA	*
F	24	Caucasian	ileocolonic	none	N
F	28	Afro-carribean	colonic	5-ASA	N
M	28	Caucasian	TI	none	N
F	21	Caucasian	colonic	5-ASA	*
F	40	Caucasian	ileocolonic	5-ASA	*
F	44	Caucasian	TI	5-ASA	Y
F	28	Caucasian	colonic	5-ASA	*
F	47	Caucasian	colonic	5-ASA	N
M	46	Caucasian	TI	5-ASA	*
M	27	Caucasian	TI, perianal	none	*
M	65	Caucasian	TI	none	Y
M	39	Caucasian	colonic	5-ASA	*
M	35	Caucasian	ileocolonic	5-ASA	*
F	38	Caucasian	TI	5-ASA	*
F	37	Caucasian	ileocolonic	none	*
M	36	Caucasian	colonic	none	N
M	42	Asian	colonic	5-ASA	N
F	61	Caucasian	colonic	5-ASA	*
M	*	Caucasian	*	none	*
M	37	Caucasian	colonic	5-ASA	*
F	27	Caucasian	ileocolonic	5-ASA	*
F	28	Asian	TI	none	*
M	47	Caucasian	TI	none	*
M	52	Caucasian	TI	5-ASA	*
M	43	Caucasian	colonic	5-ASA	*
F	33	Caucasian	ileocolonic, perianal	5-ASA	*
M	36	Caucasian	colonic, perianal	5-ASA	*
F	48	Caucasian	TI	5-ASA	*
M	57	Caucasian	ileocolonic	none	N
F	41	Asian	ileocolonic, perianal	5-ASA	*
M	45	Asian	ileocolonic	5-ASA	*
M	*	Caucasian	ileocolonic	5-ASA	*
M	20	Caucasian	colonic	5-ASA	N
F	46	Caucasian	colonic	5-ASA	N
M	56	Caucasian	ileocolonic	5-ASA	*
F	34	Asian	colonic	5-ASA	N
F	*	Caucasian	colonic	Infliximab	*
M	34	Asian	ileocolonic	5-ASA	*
F	26	Caucasian	colonic	5-ASA, Steroids	*
F	30	Caucasian	TI	5-ASA	*
F	33	Caucasian	TI	5-ASA	*
M	56	Caucasian	ileocolonic	5-ASA	*
M	*	Caucasian	TI	none	*
M	29	Caucasian	colonic	5-ASA	*
M	23	Caucasian	TI	none	N
F	40	Caucasian	ileocolonic	5-ASA	*
F	*	Caucasian	ileocolonic	none	*
M	*	Caucasian	*	*	*
F	65	Caucasian	TI	5-ASA	*
M	40	Caucasian	TI, perianal	none	*
F	68	Caucasian	ileocolonic	none	*
F	51	Caucasian	ileocolonic, perianal	5-ASA	Y
F	60	Caucasian	TI	none	*
F	36	Caucasian	ileocolonic	none	*
M	43	Caucasian	colonic	5-ASA, Steroid	*
F	23	Caucasian	ileocolonic	5-ASA	*
M	48	Caucasian	ileocolonic	5-ASA, Sildenafil	*
F	38	Caucasian	ileocolonic, fistula	none	*
F	53	Caucasian	colonic	none	N

* data not available

Supplementary Table 1
