# Supplementary figures and images for: Diminished Macrophage Apoptosis and Reactive Oxygen Species Generation after Phorbol Ester Stimulation in Crohn's Disease
Source: PLoS One. 2009 Nov 12;4(11):e7787. doi: 10.1371/journal.pone.0007787 (PMC2771353; doi:10.1371/journal.pone.0007787)

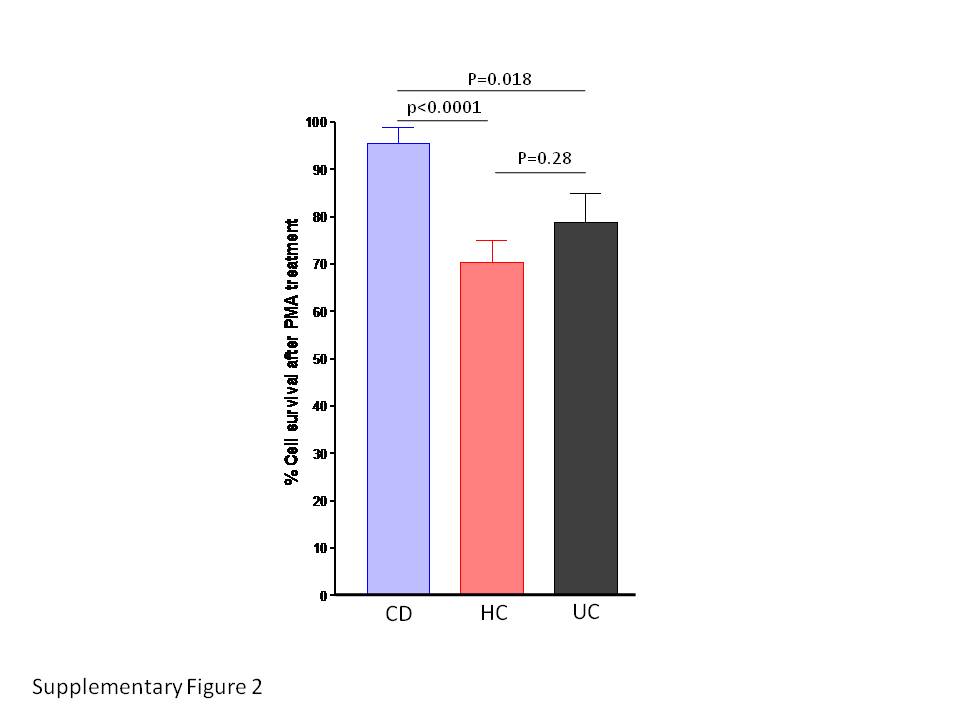

Supplement: Figure S2 — Abnormal macrophage response to PMA stimulation is specific for CD patients. Viability assay for macrophages from CD, HC and ulcerative colitis subjects following stimulation with PMA for 24 h. Data are presented as percent of untreated cells for CD (blue, n = 41), HC (red, n = 24) and UC (black, n = 13). Statistical analysis: Unpaired t-test. (0.03 MB JPG) [file pone.0007787.s003.jpg]
